# Mr. Henry, on the Treatment of Gonorrhœa

**Published:** 1803-01-01

**Authors:** William Henry

**Affiliations:** Manchester


### *To the Editors of the Medical and Physical Journal.*

GENTLEMEN,

THERE are few, I believe, of the junior practitioners of Medicine, (to whom the treatment of Venereal Cases generally falls) who must not have had frequent experience of the inefficacy of all topical applications in the virulent Gonorrhœa. The injection of a solution of white vitriol, (the zincum vitriolatum, or sulphat of zinc) in the proportion of about a grain to each ounce of water, though perhaps one of the most active and successful, I have found, in persons of irritable habit, to produce, occasionally, great pain, an increase of the discharge, and a peculiar liability to swelling of the testicles. In the year 1795, I had occasion to prepare, for a chemical experiment, a quantity of the crystalized acetite of zinc, and used it in a few cases of Gonorrhœa, that were found to resist the ordinary remedies. The success of this application exceeded my expectation, and far surpassed that of any injection, the use of which I had ever witnessed. In the winter of that year, I took with me to Edinburgh, a quantity of the acetite of zinc, and gave portions of it to various persons; among others, to my friend, Mr. George Bell, the son of Mr. Benjamin Bell, and now an eminent Surgeon in Edinburgh. What was the result of his trials of the remedy, I really forget; and, indeed, whether he ever tried it or not. But, satisfied of its superior efficacy, I have continued to use it from that period to the present, as the best application in all cases of Gonorrhœa, and very seldom with any but the most favourable issue. From ample experience, I have of late felt myself justified in recommending the remedy to several medical friends; and and the event of their practice has fully accorded with my own. Among others, Dr. Ferriar and Mr Gibson, have testified the superiority of this remedy over all others, and after the failure of various other injections.

The acetite of zinc may be prepared in any of the following modes:

1. Let zinc, which may be bought at the brazier's, under the name of spelter, be melted in an iron ladle, and poured from a considerable height into a vessel of cold water. It will thus be minutely divided, or granulated; and if the operation has been unskilfully managed, may be divided still farther by a pair of strong shears. Let the finest parts of this granulated metal be added to a quantity of distilled vinegar; or, what is still better, of the acidum acetosum of the London Pharmacopœia, diluted with six, or eight parts of water. Shake or stir the materials together frequently; and, when the acid has lost all its sourness, which will happen in a few days, let the liquor be slowly evaporated. On cooling, it will shoot into crystals of a similar colour to those of sugar of lead; or into nearly white ones, if the acidum acetosum has een the solvent.

2. For metallic zinc, the white sublimed oxide (flores zinci) may be substituted, using the same solvents as before; and the chemist will find it advantageous to employ the gritty part, which is less fit for use as an internal medicine.

3. To a solution of white vitriol (zincum vitriolatum) in six or eight times its weight of water, add a solution of the acetite of lead (cerussa acetata) in twice its weight of water, as long as any precipitation ensues, or a little longer, in order to ensure the complete decomposition of the white vitriol. Throw the whole upon a linen strainer, and wash off the soluble part by repeated affusions of distilled water; then evaporate, and crystalize as before.

The crystals of acetite of zinc, may be obtained by very cautious and slow evaporation, of a distinct and regular form; but, for medicinal use, this nicety is quite unnecessary.

For the purpose of an injection, I generally use eight or ten grains, dissolved in four or six ounces of water, or, what is much better, in the same quantity of a thin mucilage of quince seeds, or of decoction of linseed, or of barley. The strength may be increased or diminished, as may be found expedient; but such a proportion as excites a slight smarting, I consider as rather favourable to the cure.

A solution

A solution of the salt, in smaller proportion than that above assigned, in rose water, forms an excellent eye-wash. The medicine may also be exhibited internally, (as my father has frequently done) in all cases in which the calcined zinc has hitherto been employed, and with more certain efficacy. In the dose of from 5 to 10 grains, it affords a safe emetic, which operates speedily.

It is but justice to Mr. Benjamin Bell to acknowledge, that since I began to use the acetited zinc in Gonorrhœa, I have observed, in the Appendix to his Treatise on the Venereal Disease, a formula for preparing a similar injection, extemporaneously, by the mixture of the sulphate of zinc with the acetite of lead. I apprehend, however, that it will be found much more convenient to keep the acetite of zinc ready prepared; that the strength of the solution will be more certainly regulated; and its freedom, from contamination with white vitriol, better secured. I am, &c.

*Manchester, Dec.* 5, 1802. WILLIAM HENRY.

P. S. The acetite of zinc, when administered internally, should be that which has been prepared by the direct combination of the metal, or of its oxides, and not by double affinity; as deleterious effects may be produced by a redundancy of the acetite of lead.

